# Effect of Decompressive Craniectomy on Intracranial Pressure Waveforms and Vascular Reactivity: A Systematic Scoping Review

**DOI:** 10.1089/neur.2024.0046

**Published:** 2024-10-02

**Authors:** Tommaso Rochat, Stefan Yu Bögli, Erta Beqiri, Hervé Quintard, Marek Czosnyka, Peter Hutchinson, Peter Smielewski

**Affiliations:** ^1^ Brain Physics Laboratory, Division of Neurosurgery, Department of Clinical Neurosciences, University of Cambridge, Cambridge, United Kingdom of Great Britain and Northern Ireland.; ^2^ Division of Intensive Care Medicine, Geneva University Hospital, Geneva, Switzerland.; ^3^ Division of Neurosurgery, Addenbrooke’s Hospital, Cambridge University Hospitals NHS Foundation Trust, Cambridge, United Kingdom of Great Britain and Northern Ireland.

**Keywords:** decompressive craniectomy, ICP waveforms, PRx, slow waves, TBI

## Abstract

Decompressive craniectomy (DC) primarily aims at decreasing intracranial pressure (ICP) by allowing for the brain tissue to expand. However, it is uncertain to what extent DC impacts the transmission of vasogenic slow waves and thus the validity and utility of the pressure reactivity index (PRx). The purpose of this systematically performed scoping review is to assess the current knowledge of the impact of DC on ICP waveforms and measures of vascular reactivity. This scoping review considered studies including patients over 18 years old suffering from acute brain injuries (ABIs), who underwent secondary DC and had a perioperative (pre/post-DC) recording of ICP or waveform analysis. A search was conducted in EMBASE, PubMed, Web of Science, Scopus, and Medline from November 2023 till January 2024, yielding 787 studies. Duplicated studies were automatically removed, and two researchers independently screened the remaining studies. After examining 586 titles and abstracts, 38 full-text studies were assessed for eligibility, and 4 studies were included in the final review. The review suggests that cerebrovascular reactivity and slow waves are altered after DC, with positive PRx values and reduced slow power. One study suggested that the nature of slow waves and interactions is on the whole largely preserved. However, the findings should be interpreted with caution due to methodological limitations and the low number of studies.

## Introduction

Intracranial hypertension is common in patients with head injury. The primary goal of treatment is to lower the intracranial pressure (ICP) and to ensure adequate Cerebral perfusion pressure (CPP), guided, if available, by cerebrovascular reactivity monitoring.^1–4^ Monitoring of cerebral autoregulation is by now understood to be valuable for prognostication and potentially for individualizing CPP management in order to achieve augmentation of outcome in patients with moderate to severe traumatic brain injury (TBI) in adults.^5–8^ Several studies have underlined the robust correlation between cerebrovascular reactivity metrics derived from ICP and overall outcome.^5,9,10^ The pressure reactivity index (PRx), which correlates slow waves of mean arterial pressure (MAP) with vasogenic slow waves of intracranial pressure, is the most validated and widely used measure of cerebrovascular reactivity in moderate or severe TBI.^1,8,11,12^

Despite careful clinical management, brain swelling can progress in patients with head injury, obstructing cerebral blood flow and ultimately leading to refractory increases in ICP, to an autoregulatory vasodilatory cascade leading to further increases in ICP and subsequent oedema.^13^ In situations where other methods of reducing ICP are ineffective (first and second tier ICP management, as defined by the Brain Trauma Foundation guidelines^2^), decompressive craniectomy (DC) is considered. DC primarily aims at decreasing ICP by allowing for the brain tissue to expand. Its effectiveness in reducing ICP values and influence on mortality and neurological outcomes are well-established.^4,14^ However, the treatment of acute brain injury (ABI) does not end with DC; prevention of secondary brain injury in the post-operative period is also pivotal.^15^ The loss or impairment of the cerebrovascular autoregulatory response is associated with the development of secondary brain injury. To optimize target therapy, it is desirable to monitor the autoregulatory reserve in these patients.

DC intuitively increases cerebral compliance and therefore introduces circumstances where the normal waveforms may be disrupted, potentially rendering secondary metrics based on waveform analysis invalid.^16,17^ It is uncertain how and to what extent DC impacts the transmission of vasogenic slow waves, and therefore the validity and utility of PRx after DC remain unclear. To date, only a small number of studies have examined changes in these variables before and after DC.

The purpose of this systematically performed scoping review was to assess the current understanding of the impact of DC on ICP waveforms and measures of vascular reactivity to determine the validity and utility of these parameters post-craniectomy.

### Review question

The objective of this systematic scoping review is to provide a description of the extent and type of evidence regarding the effects of DC on cerebrovascular reactivity and slow waves. Using the population, concept, and context framework, the following specific question was formulated:

What is the evidence for changes in ICP waves and vascular reactivity after DC in adults and is PRx still a reliable monitoring technique?

## Methods

This scoping review was conducted in accordance with the Joanna Briggs Institute methodology for scoping reviews.

### Participants

This scoping review considered studies including patients over 18 years old suffering from ABIs, who underwent secondary DC to treat ongoing refractory ICP and had a perioperative (pre-/post-DC) recording of ICP or waveform analysis in addition to the basic physiological parameters. All studies in the pediatric population (<18 years) were excluded.

### Context

This scoping review did not consider the specific race, gender, or geographic location of participants in the selected studies. Given that the anatomy and pathophysiology of TBI within the pediatric population differ substantially from those of their adult counterparts, exclusion was determined solely by participant age, with only studies conducted in the adult population (>18 years) being included.

### Types of sources

This scoping review considered both experimental and quasi-experimental study designs including randomized controlled trials, non-randomized controlled trials, before and after studies, and interrupted time-series studies. In addition, analytical observational studies including prospective and retrospective cohort studies, case–control studies, and analytical cross-sectional studies have been considered for inclusion. This review considered descriptive observational study designs including case series. This study has not considered individual case reports and descriptive cross-sectional studies for inclusion.

Text and opinion articles have not been considered for inclusion in this scoping review.

### Search strategy

An initial search in EMBASE, PubMed, Web of Science, Scopus, and Medline was undertaken from November 2023 till January 2024, aimed at identifying published studies in the adult population to obtain the most updated evidence on the subject. Studies published in languages other than English were excluded. Studies that did not contain data recordings of ICP waveform analysis or PRx pre- and post-DC were also not included. Studies containing patients with DC were included independently from the etiology of brain injury. A detailed search strategy for all databases is contained in Supplementary Appendix Figure SA1.

### Study/source of evidence selection

The initial search yielded 787 studies. All identified citations were uploaded into Covidence, and 201 duplicated studies were removed automatically. Studies were screened independently by two researchers (T.R. and S.B.). Any disagreements that arose between the reviewers at each stage of the selection process were resolved through discussion, or with an additional reviewer (P.S.).

After examining 586 titles and abstracts for inclusion, 547 irrelevant studies were removed, 39 full-text studies were assessed for eligibility, and 35 studies were excluded for reasons described in Supplementary Appendix Figure SA1.

The results of the search and the study inclusion process are reported in full in the final scoping review and presented in a Preferred Reporting Items for Systematic Reviews and Meta-analyses extension for scoping review flow diagram.^18^

### Data extraction

Data has been extracted from articles included in the scoping review by two independent reviewers (T.R. and S.B.) using the data extraction tool (Covidence) developed in the protocol and modified after initial review of the articles to present data in the clearest manner. The data extracted include specific details about the participants, concept, context, study methods, and key findings relevant to the review question/s. Any disagreements between the reviewers were resolved through discussion or with an additional reviewer.

## Results

This review includes four studies, consisting of two prospective observational and two retrospective studies.^17,19–21^ They were conducted over various periods ranging from 1991 to 2020, across the United Kingdom, Singapore, the Netherlands, and the European consortium Center-TBI.

In total, 78 patients were included in the reviewed studies, and three out of the four studies focused solely on individuals with TBI. Only one study also considered non-TBI patients (Wang et al.). In three studies, the type of DC was specified and included unilateral or bifrontal craniectomy. All patients who underwent DC intervention had their ICP, CPP, and MAP values reported before and after the procedure, except for one study. All studies examined PRx values, and one of them additionally assessed RAP, Pbt02, and slow waves. In two studies, high-resolution data were analyzed. Details of the included studies are shown in Table 1.

**Table 1.** Characteristics of Included Publications

Study ID	Country	Study design	Start date	End date	N° of patients	Pathology	GCS	Age	ICP monitoring	Type of DC	PRx pre-DC	PRx post-DC	*p* value	RAP pre-DC	RAP post-DC	*p* value	Slow waves pre-DC	Slow waves post-DC	*p* value
Wang, 2006	Singapore	Observational prospective	April 2001	October 2002	25	17 TBI; 4 ICH; 4 SAH	9 (IQR 7–10)	50 ± 17	Parenchymal	Bifrontal or Unilateral	Not Given	0.15 ± 0.25	N/A	N/A	N/A	N/A	N/A	N/A	N/A
Chi, 2008	Singapore	Observational prospective	January 2005	January 2006	16	TBI	5 (IQR 3–7)	38 ± 15	Parenchymal	Bifrontal or Unilateral	(G1) 0.34 ± 0.2 (G2) 0.31 ± 0.2	(G1) 0.25 ± 0.2 (G2) 0.19 ± 0.1	0.02 0.001	N/A	N/A	N/A	N/A	N/A	N/A
Timofeev, 2008	United Kingdom	Observational retrospective	2000	2005	27	TBI	74% (GCS <8) 26% (GCS >9 < 12)	35 (IQR 16–55)	Parenchymal	Bifrontal or Unilateral	−0.03 [−0.13; 0.06]	0.14 [0.12; 0.22]	0.01	0.4 [0.33; 0.68]	0.14 [0.12; 0.22]	<0.001	4.9 [0.7; 7.4]	4.9 [0.7; 7.4]	0,017
Zeiler, 2020	United Kingdom; Netherlands	Observational retrospective	January 2015	December 2017	10	TBI	4 (IQR 1–5)	34 (SD 18.1)	Parenchymal or Ventricular	Not given	0,07 ± 0.234	−0,007 ± 0.156	853	N/A	N/A	N/A	Not given	Reported as unchanged	Not given

DC, decompressive craniectomy; G1, group 1; G2, group 2; GCS, Glasgow Coma Scale; ICH, intracranial hemorrhage; IQR, interquartile range; PRx, pressure reactivity index; SAH, subarachnoid hemorrhage; TBI, traumatic brain injury.

Below we present the salient features of the included studies.

Wang et al. carried out a prospective observational study from April 2001 to October 2002, including patients with ABIs caused by various etiologies who underwent DC. They assessed ICP, CPP, and PRx evolution for 72 h post-DC. A total of 25 DC patients were evaluated and compared to a control group (six patients) receiving medical therapy solely. Four patients underwent bifrontal craniectomy, while 21 received unilateral craniectomy. The ICP, MAP, CPP, and PRx time series data was divided into 6-h no-overlapping intervals post-DC, and mean values were calculated over each interval. Their results demonstrate that post-DC, all patients exhibited a positive PRx (0.3 ± 0.21), suggesting an altered cerebrovascular reactivity despite a clear decrease in ICP and retained CPP values above 70 mmHg. A trend toward improvement was observed during the 72-h period (mean PRx at 72 h 0.15 ± 0.25; *p* = 0.012), with 16% of patients returning to a negative PRx.

Timofeev et al. conducted a retrospective observational study from 2000 to 2005. DC was performed bifrontally in 78% of cases and unilaterally in the remaining cases. The study included 17 TBI patients, and data analysis focused on ICP, ICP waveforms, MAP, CPP, RAP, and PRx values. The compensatory reserve index (RAP) and PRx were both calculated using a linear correlation between 40 consecutive time-averaged (6 or 10 sec) data points.

All parameters were averaged for the total periods before and after the operation. Additionally, averages around other time points were evaluated (24 and 3 h before DC; 3, 24, 48, and 72 h after DC) to explore the time profiles.

Overall, the study revealed that the median values of ICP reduced significantly from a median value of 21.1 to 15.7 mmHg (*p* < 0.01), slow waves reduced from 4.9 to 1.8 (*p* < 0.017), and RAP decreased from 0.4 to 0.14 (*p* < 0.001) after DC. The CPP values showed hardly any change. Interestingly, the pressure reactivity index (PRx) exhibited a sustained rise toward more positive values after DC, although the median value remained below the threshold of 0.2 (−0.03 to 1.8; *p* = 0.01). The researchers demonstrated that the percentage of time PRx exceeding the threshold of 0.2 increased almost twofold after DC (from 23.9% to 42.8%). Concerning outcome, post-operative PRx values remained significantly higher in patients with unfavorable outcomes.

In 2008, Chi Long Ho and colleagues conducted a prospective observational study to assess the impact of DC on cerebral oxygenation, cerebrovascular reactivity, and cerebral microdialysis.

The study included 16 TBI patients (81% male) who underwent secondary bifrontal or unilateral DC (50% each).

Hourly recordings of ICP, MAP, and CPP values, as well as Pb02 and microdialysis, were taken. PRx was calculated as the correlation coefficient between the previous 30 samples, averaged over a period of 10 sec.

Patients were divided into two groups based on their GOS scores.

Analyses were conducted for each group, and the results demonstrate a significant reduction in ICP after DC for both groups, with a CPP above the fixed guideline conform threshold.

The PRx displayed impaired autoregulatory values (>0.3) in both groups before DC, with decreasing values after the operation. A greater reduction was observed in the group with the better outcome. Nonetheless, it is important to note that the threshold value was set at 0.3 and no internationally accepted threshold value exists at this moment. Overall, neither the favorable nor the unfavorable outcome group achieved values of preserved autoregulation. Upon examining the post-DC values in both groups, PRx values were >0.2 (from 0.31 to 0.19) in group 2 and from 0.34 to 0.25 in group 1. No between-group comparisons were made to assess whether these differences were significant.

Zeiler et al. conducted a retrospective analysis within the CENTER-TBI cohort to determine whether the properties of the vascular reactivity metric and the slow-wave relationship are affected by DC. They included 10 ICU patients with high-resolution data for a period of at least 24 h before DC and 48 h after. The data were analyzed by pooling them for each time period around the secondary DC; in addition, the optimal ARIMA structure for PRx and the ICP–MAP slow-wave relationship was explored using VARIMA modeling and Granger analysis.

The study shows a statistically significant reduction in ICP after DC (from 15.9 mmHg SD 5.2 to 11.1 mmHg SD 4.0; *p* = 0.043), as well as MAP and % of time with ICP >22 mmHg (12% SD 16.9 to 1.3% SD 2.4; *p* = 0.002). They also reported that PRx and % of PRx above threshold were not affected by DC (0.070% SD 0.23 to −0.007% SD 0.15; *p* = 0.853). Similarly, the time structure of PRx before and after DC did not change, nor did the slow-wave ICP–MAP relationship.

## Discussion

The data analyzed and presented in the articles of this systematic scoping review indicate that the effect of DC on brain parameters, particularly PRx, remains controversial.

### RAP and slow waves

RAP is defined as the moving Pearson correlation coefficient between mean ICP and pulse amplitude of ICP (AMP), usually evaluated over 300 sec period.^22^ Positive RAP values close to zero indicate a preserved compensatory reserve. However, values approaching one indicate an impaired compensatory reserve (i.e., steep, exponential relationship between volume and pressure). The RAP and amplitude of slow waves were reported in one and two studies, respectively. Notably, Timofeev et al. found a decrease in both parameters after DC. Furthermore, an analysis of the slow arterial blood pressure (ABP) waves within the same cohort was missing, thus their transmission to ICP cannot be judged. RAP, on the contrary, decreased after DC providing evidence for an improved compensatory reserve.

### PRx

Overall, studies showed either: (1) no difference in pre- vs. post-DC PRx; (2) an increase of PRx after DC; and (3) an increase of duration spent above PRx of 0.2. No study showed an improvement of PRx returning it to the working autoregulatory range of below −0.2 to 3. While Chi Long et al. showed a relative decline of PRx post-DC, the values remained above 0.2, thus indicating impaired autoregulation pre and post-DC. It is also noteworthy that Wang et al. report a temporal progression of improvement amongst the 72 h post-operative with 16% of patients having PRx values close to zero or turning negative. It is unclear if this trend is due to the DC or the temporal dynamics of TBI with PRx commonly improving with longer time monitored.

Several hypotheses could be evaluated to explain the change in PRx after DC. One possible explanation for such a pronounced alteration in pressure reactivity might be the formation of a post-decompression hyperemia with linked vasoparesis. According to certain reports, CBF and brain tissue oxygen values might rise up following a DC, reaching levels much greater than anticipated.^15,23^ However, it remains to be established whether modified vascular reactivity is linked to the cerebral oedema subsequent to DC.^24^

Furthermore, after DC, the prerequisites for the validity of PRx calculation may be infringed. PRx relies on the adequacy of transmission of volume changes from ABP to ICP and thus volume conservation, which is altered after DC. DC consistently leads to a decrease of ICP with increased compliance, as shown by the increased compensatory reserve. Consequently, PRx may no longer provide valid information about cerebrovascular pressure reactivity. However, Zeiler et al. argued that in their small cohort of 10 patients’ statistical properties of PRx time series before and after the DC seemed not to be visibly affected, though no formal tests of significance of this finding were performed. Similarly, using vector ARMA analysis of mean ABP and ICP time series used for the calculation of PRx, they showed similar-looking transmission characteristics from ABP to ICP before and post-DC. Again, here the comparisons were visual, based on impulse response functions generated directly from the VARMA models fitted to the data. Finally, they also showed, in another metric derived from the VARMA model, Granger causality, that there was a significant effect of ABP acting on ICP, and this did not change after DC. Thus, although all these results are certainly suggestive, the study cannot be treated as conclusive, and the question of whether or not the assumption of PRx is affected by DC still remains open.

Considering the outcome, only two studies described a difference in PRx post-DC comparing outcome. While higher PRx values were still associated with unfavorable outcome post-DC (GOS at 6 months), it remains unclear whether its discriminatory value changed compared to pre-DC as no analysis thereof was presented.

### Limitations

This scoping review has limitations. First, the limited number of articles and the quality of the data do not allow for any further statistical analysis. Moreover, the majority of patients suffered TBI, and the type of DC was diverse allowing for little generalizability. Finally, the quality of the interpretation and validity of the results are further diminished as only two studies used high-resolution data in their analysis.

## Conclusion

In conclusion, the articles in this review intuitively show that RAP decreases after DC, while PRx values and slow waves are reported to have contrasting results. However, methodological limitations and low number of studies should be considered when interpreting these findings.

Nevertheless, the prevalence and clinical relevance of these parameters warrant further investigations to confirm their validity after DC.

## Supplementary Material

Supplementary Appendix Figure SA1

## Abbreviations Used

ABI: Acute brain injury
ABP: Arterial blood pressure
ARIMA: Autoregressive integrated moving average
CPP: Cerebral Perfusion Pressure
DC: Decompressione Craniectomy
GCS: Glasgow come scale
ICP: Intracranial pressure
MAP: Mean Arterial Pressure
PRx: Pressure reactivity index
RAP: Compensatory reserve index
TBI: Traumatic brain injury
VARIMA: Vector autoregressive integrated moving average
