# Supplementary figures and images for: Effect of Decompressive Craniectomy on Intracranial Pressure Waveforms and Vascular Reactivity: A Systematic Scoping Review
Source: Neurotrauma Rep. 2024 Oct 2;5(1):903–9. doi: 10.1089/neur.2024.0046 (PMC11512082; doi:10.1089/neur.2024.0046)

## Effect of Decompressive Craniectomy on ICP wave forms

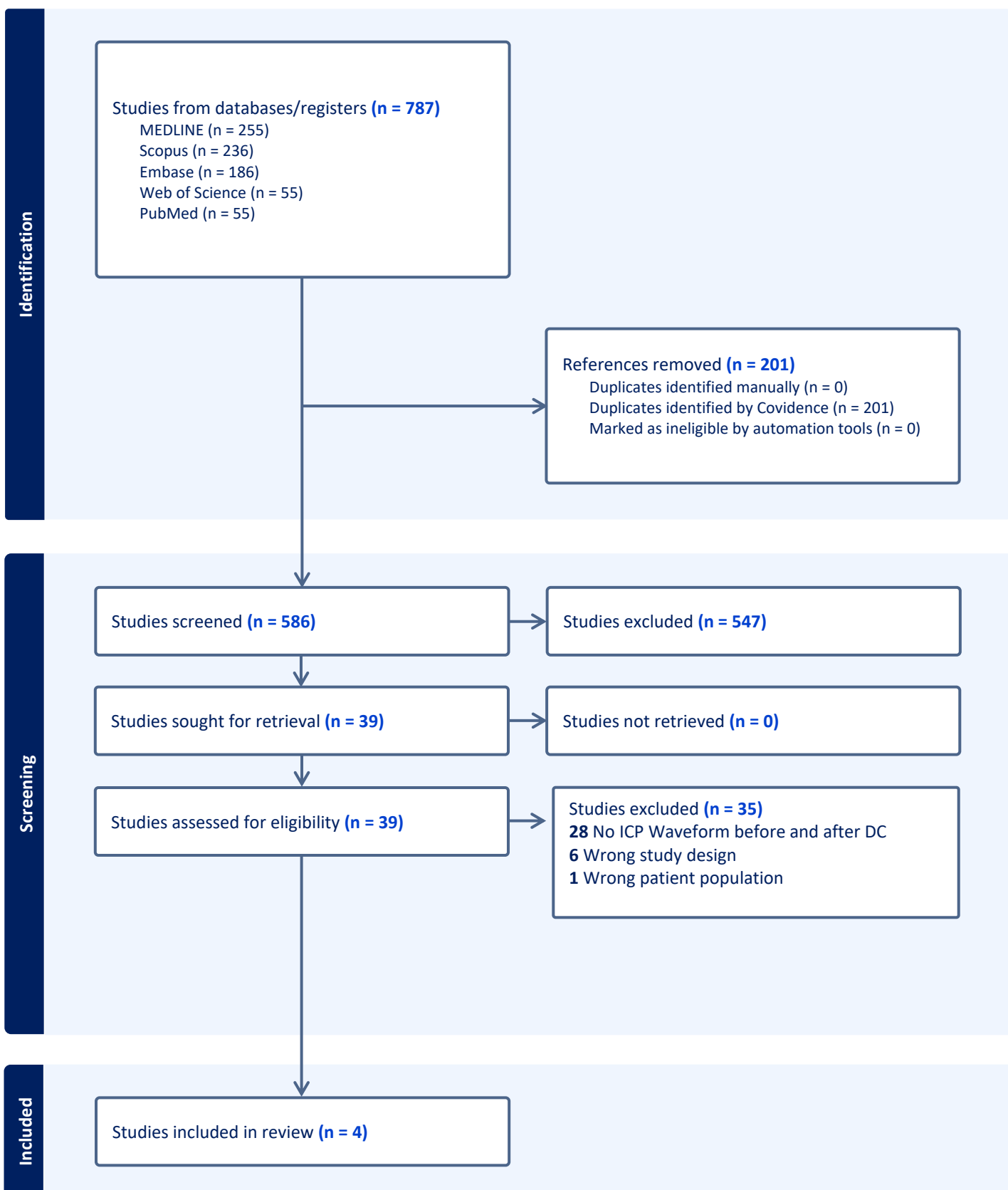

Supplement: Supplementary Appendix Figure SA1 [file neur.2024.0046_supp_appfigsa1.pdf]
